# Virulence-associated variants in *Cryptococcus neoformans* sequence type 93 are less likely to be associated with population structure compared to independent rare mutations

**DOI:** 10.1128/spectrum.01709-24

**Published:** 2024-11-27

**Authors:** Katrina M. Jackson, Kisakye Diana Kabbale, Marissa Macchietto, David Meya, Peter Tiffin, Kirsten Nielsen

**Affiliations:** 1 Department of Microbiology and Immunology, University of Minnesota, Minneapolis, Minnesota, USA; 2 Pathogen and Microbiome Institute, Northern Arizona University, Flagstaff, Arizona, USA; 3 Infectious Diseases Institute, College of Health Sciences, Makerere University, Kampala, Uganda; 4 African Center of Excellence in Bioinformatics and Data Intensive Sciences, Kampala, Uganda; 5 Minnesota Supercomputing Institute, University of Minnesota, Minneapolis, Minnesota, USA; 6 Department of Plant and Microbial Biology, University of Minnesota, St. Paul, Minnesota, USA; 7 Department of Biomedical Sciences and Pathology, Virginia Tech, Blacksburg, Virginia, USA; University of Wisconsin-Madison, Madison, Wisconsin, USA

**Keywords:** *Cryptococcus neoformans*, linkage disequilirium, population genetics, molecular genetics, virulence determinants, mycology, DNA sequencing

## Abstract

*Cryptococcus neoformans* is a pathogenic yeast that is the causative agent of cryptococcal meningitis. While it is well known that the genotype of *C. neoformans* impacts patient outcomes, the reason for this association has not been well elucidated. In this study, we examined the relationship between two subpopulations in the sequence type 93 clade of *C. neoformans*: ST93A and ST93B. We found extensive linkage disequilibrium (LD) among the single nucleotide polymorphisms (SNPs) that differentiate ST93A from ST93B. We also found differences in the extent of linkage among SNPs within each subpopulation; LD was more extensive within ST93B than ST93A. SNPs associated with virulence were in long-range linkage disequilibrium with less frequency than recurrent SNPs not associated with virulence. We investigated the karyotype of ST93A and ST93B using contour-clamped gel electrophoresis and long-read sequencing and found that the extensive long-range linkage was not due to chromosomal rearrangements. Overall, we found that the two subpopulations in ST93 are driven by SNPs in LD. We additionally found that recurrent SNPs associated with virulence were less frequently evolutionarily linked and were two times more likely to be independent, congruent mutations rather than tied to phylogeny.

**IMPORTANCE:**

*Cryptococcus neoformans* is an important pathogen that is widely distributed and ubiquitous in the environment. The majority of the human population has a latent, controlled infection suggesting that *C. neoformans* is uniquely adapted to cause infection. In spite of this, the reason *C. neoformans* is a pathogen remains unknown; interestingly, most environmental isolates are avirulent but are genetically very similar to disease-causing virulent isolates. Recent evidence from genome-wide association studies shows that small mutations in key virulence-associated genes are associated with the virulence of specific isolates. The data presented here provide an evolutionary framework for those small mutations. The mutations that impact disease are not being collected over long-term evolution. The mutations may instead occur independently during infection. Identifying these genes that are more likely to be mutated during infection will be fundamental for understanding *C. neoformans* virulence.

## INTRODUCTION

*Cryptococcus neoformans* is a medically important human fungal pathogen that is widely distributed in the environment. Infections are environmentally acquired and it is hypothesized that up to 95% of individuals worldwide have a controlled infection in the lungs (1, 2). When a person becomes immunocompromised, most commonly due to advanced HIV infection but also in patients undergoing solid organ transplant and chemotherapy, the yeast cells can disseminate from the lungs and cause disease (3). An estimated 15–19% of all AIDS-related deaths are from cryptococcal meningitis and 180,000 people suffering from advanced HIV infection succumb to *C. neoformans* infection annually (4, 5). In 2022, the WHO declared *C. neoformans* to be a critical priority fungal pathogen (6).

The global *C. neoformans* population is split into three major lineages: VNI, VNII, and VNB (7). Of those, both VNI and VNII are largely clonal and globally distributed while VNB is diverse. Each lineage is further subdivided into sequence types (STs) based on a multilocus sequence typing (MLST) scheme (8, 9). Studies in the last decade revealed that both genetic lineage and sequence type are associated with patient survival and disease (7, 10–18). Further works showed that when mice are infected with clinical isolates, mouse mortality is similar to patient mortality, and changes in patient survival are due in part to differences between isolates (19). However, the reasons for the link between genotype and patient outcome remain poorly understood.

Recent genomic studies investigated the link between small genetic variations and virulence (20–22). Desjardins et al. compared clinical and environmental isolates and identified differences in stress response and virulence-related genes (20). We showed, in a closely related population of clinical isolates from the ST93 clade, that small variations [single nucleotide polymorphisms (SNPs), insertions, and deletions] were associated with changes in patient immune response and disease outcome (21). Similarly, Sephton-Clark et al. found in a diverse collection of clinical isolates that small variations were associated with changes in fungal burden in patients (22). Overall, these studies suggest that small variations in key virulence-associated genes of *C. neoformans* associate with strain-specific virulence.

In our previous study (21), we found that ST93 had two subpopulations, ST93A and ST93B. In this study, we examine the ST93A and ST93B subpopulation split in more detail. We performed a linkage disequilibrium (LD) analysis and showed that the linkage groups were highly segregated between the two subpopulations. We found that SNPs associated with human infection phenotypes were twice as likely to be rare independent mutations rather than being evolutionarily linked. Finally, we used contour-clamped gel electrophoresis and Oxford Nanopore long-read sequencing to show that the linkage segregation was not the result of structural variations between chromosomes.

## RESULTS

### ST93 has two subpopulations and SNPs associated with virulence are in both subpopulations

In a previous study, we performed Illumina whole genome sequencing on 38 clinical isolates from the ST 93 clade of *C. neoformans* (21). Each clinical isolate had matching patient data, including patient survival, immune response, and clinical parameters of disease. Gerstein et al.’s analyses of those data revealed that ST93 formed two distinct subpopulations: ST93A and ST93B. These studies also performed a genome-wide association study (GWAS) and identified 207 point mutations, insertions/deletions, and other genetic derivatives compared to the H99 reference genome, collectively referred to as SNPs, that were significantly associated with the human infection data (Table S1). Interestingly, we observed that SNPs associated with virulence were found in both subpopulations, despite the clear subpopulation differentiation (21). To further understand this, we further investigated the SNPs leading to the ST93 A versus B subpopulation differentiation.

### ST93B has a higher percentage of SNPs in long-range linkage disequilibrium than ST93A

Gerstein et al. (21) found that the ST93 clade of *C. neoformans* had 652 SNPs that were predicted to have an effect on either gene regulation or protein amino acid sequence and were polymorphic within the ST93 population. Of those, 434 SNPs were segregated among both ST93A and ST93B isolates (Table 1). There were 212 SNPs segregating only among ST93A isolates and 218 SNPs segregating only among ST93B isolates.

**TABLE 1:** Summary of linkage disequilibrium patterns in ST93, ST93A, and ST93B

	ST93	ST93A	ST93B
Total SNPs^*a*^	652	434	440
SNP loci*^b^*	405	309	282
SNP loci in long-range linkage^*c*^	184	56	107
Percent SNP loci in long-range linkage	45.43%	18.12%	37.94%
Number of long-range linkage groups	19	6	15
SNPs associated with human infection phenotypes	207	162	200
SNP loci associated with human infection phenotypes	182	141	175
SNP loci in long-range linkage	44	7	42
Percent of SNP loci associated with human infection phenotypes in long-range linkage	24.18%	4.96%	24.00%
Number of long-range linkage groups	5	1	4

^*a*^ Includes variants that are not associated with human infection phenotypes.

^*b*^ SNP loci are the number of variants when treating SNPs in less than 13,000 bp apart as a single variant.

^*c*^ SNPs in LRLD, where SNPs in SRLD are treated as a single variant.

**Fig 1:**
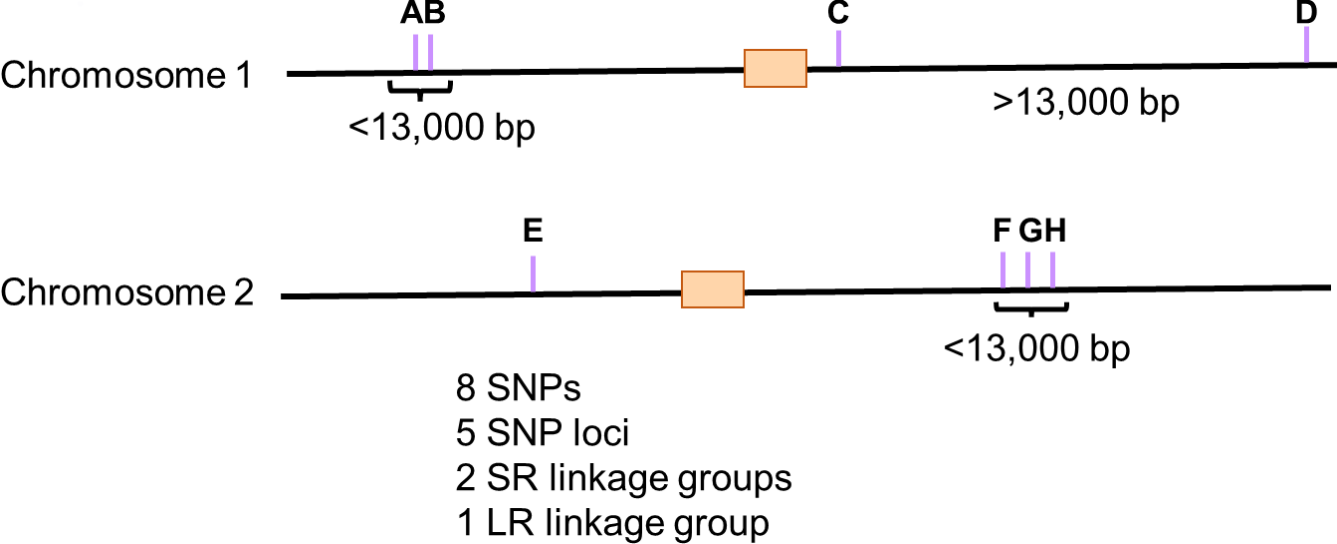
Model displaying a linkage group containing SNPs in SRLD and LRLD. SNPs A and B and SNPs F, G, and H are in short-range linkage disequilibrium and are each defined as one SNP loci, as they are less than 1 cM apart in genomic distance. The linkage group shown here contains SNP loci A, SNP loci F, and SNPs C, D, and E. SNPs closer than 1 centimorgan (cM) are collapsed into one SNP loci.

We hypothesized that the population subdivision is likely responsible for some SNPs that segregate only among ST93A or ST93B isolates. To investigate this, we performed a linkage disequilibrium analysis to identify pairs of SNPs that were found together more often than expected under free recombination. We used the genomic distance between two or more SNPs to separate SNPs that were in linkage disequilibrium into two groups. We considered SNPs to be in long-range linkage disequilibrium (LRLD) if they were more than 1 centimorgan (cM) apart in genetic distance: 13,000 bp in *C. neoformans* (23). We considered SNPs to be in short-range linkage disequilibrium (SRLD) when less than 13,000 bp apart in genomic distance (Fig. 1). We were most interested in SNPs in LRLD, as these would be more informative about population structure. Thus, multiple SNPs in SRLD within 13,000 bp were called a single “SNP locus,” regardless of the number of SNPs in that region.

**Fig 2:**
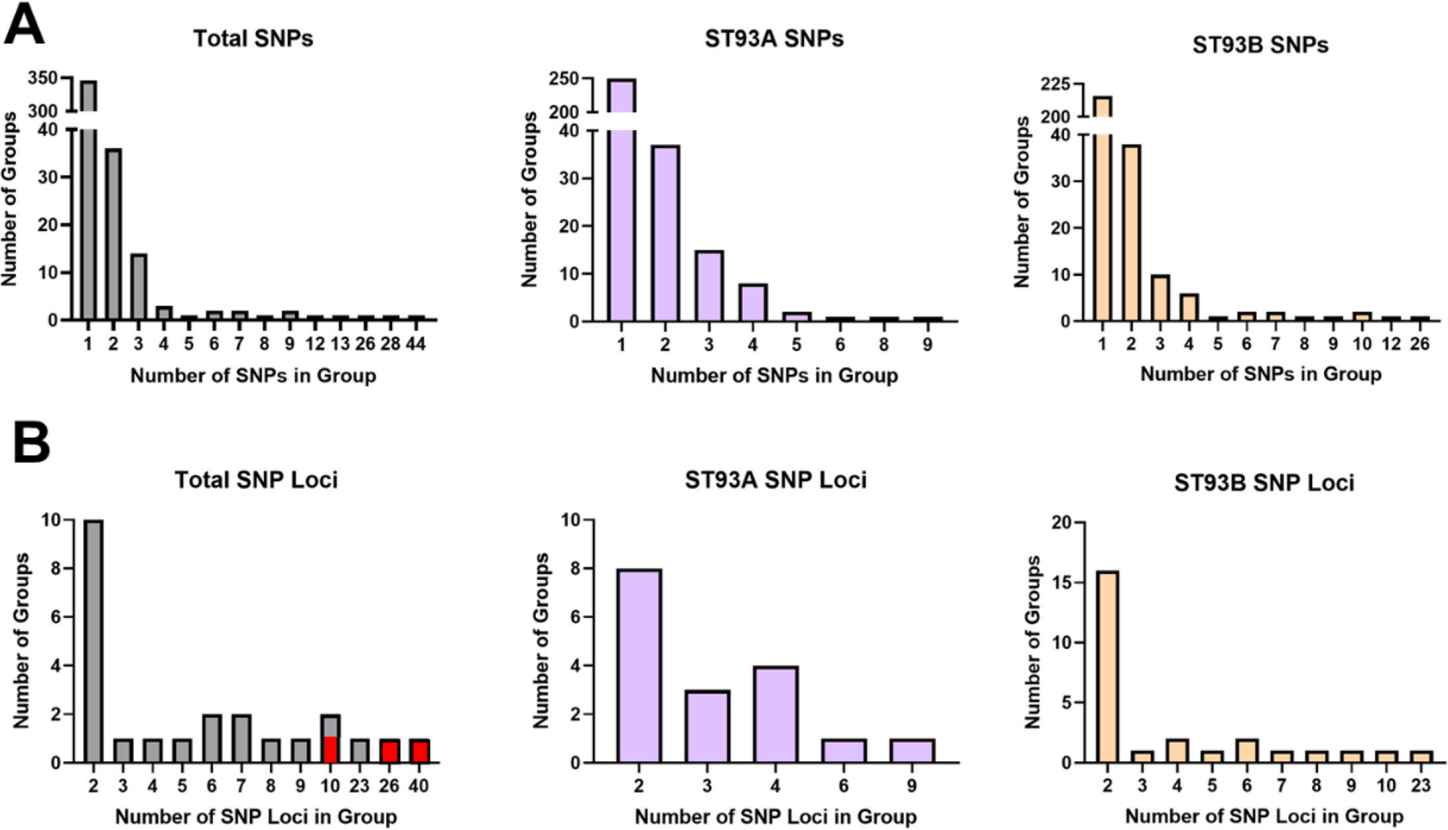
The size of linkage groups varies across ST93A and ST93B. (A) The vast majority of SNPs are singletons or in small groups of two or three SNPs, but large groups of up to 44 linked SNPs are also present. ST93A has fewer linkage groups of smaller size than ST93B. (B) While there are fewer long-range linkage groups, they have more SNPs than the short-range linkage groups. ST93A has fewer long-range linkage groups than ST93B. The red bars indicate linkage groups that span both subpopulations, differentiating A and B, and are therefore not included in the subpopulation-specific linkage groups.

Of the 652 SNPs segregating among the ST93 isolates, most SNPs (346) were not in LD with other SNPs and 122 were in SRLD (59 SRLD linkage groups), resulting in 405 SNPs and SNP loci (346 singleton SNPs and 59 SNP loci). 184 SNPs, or 45.4%, were in LRLD (19 LRLD linkage groups, Table 1). Most linkage groups were comprised of only 2 SNPs whereas the largest linkage group was comprised of 44 SNPs (Fig. 2).

Next, we looked at SNPs found either in ST93A or ST93B. Among the ST93A isolates, 309 SNPs and SNP loci were specific to the ST93A population; 18.1% of those SNPs were in LRLD (Table 1), significantly fewer than were in LRLD among all ST93 samples (*P* < 0.0001). Among the ST93B isolates, 282 SNPs and SNP loci were segregating, 37.9% of which were in LRLD (Table 1), significantly fewer than among all ST93 isolates (*P* = 0.0068), but twofold more than in ST93A.

We next wanted to understand the size distribution of linkage groups in each of the three populations. We first examined the distribution of all SNPs in the three populations by linkage groups. Most SNPs were not linked (ST93 = 346 SNPs, ST93A = 250 SNPs, and ST93B = 216 SNPs) and while groups of two and three were common, we identified large groups of SNPs contained within linkage groups (ST93 = 44 SNPs, ST93A = 9 SNPs, and ST93B = 26 SNPs) (Fig. 2). When we removed all singleton SNPs and SNPs in SRLD and repeated the analysis, we saw that linkage groups containing two SNPs were the most common but linkage groups with more SNPs tended to be primarily made up of SNPs in LRLD. Large groups of SNPs in ST93 included those linkage groups that segregated between ST93A and ST93B. ST93B had more SNPs in large groups than ST93A and the largest group in ST93B had more SNPs than ST93A (ST93 = 40 SNPs, ST93A = 9 SNPs, and ST93B = 23 SNPs) (Fig. 2). These results are consistent with phylogenetic evidence showing that ST93B is a distinct population with a more structured population.

### SNPs associated with human infection phenotypes are less likely to be in linkage disequilibrium

We next compared the extent of linkage disequilibrium among SNPs associated with human infection phenotypes (21) to all the SNPs in the population. In our previous study, we performed a GWAS and used logistic regression to compare the 652 SNPs in the *C. neoformans* clinical isolates to various aspects of patient clinical data, including patient survival, immune response, and clinical parameters of disease such as CD4^+^T cell count or HIV viral load. We found that 207 SNPs were associated with at least one of these phenotypes (Table S1).

Interestingly, only 24.2% of SNPs associated with human infection phenotype were in LRLD (Table 1), significantly lower than the 45.4% of all SNPs (*P* < 0.0001). All human infection phenotype SNPs in linkage were associated with changes in patient IL-2 levels (Table S2); in our previous study, we found that IL-2 levels were significantly higher in the ST93A subpopulation compared to ST93B (21). When we examined subpopulation-specific SNPs, we saw similar results. In the ST93A subpopulation, only 4.96% of SNPs associated with human infection phenotypes were in LRLD, compared to 18.1% of all SNPs present in the ST93A subpopulation (*P* value < 0.0001) (Table 1). As for SNPs in the ST93B subpopulation, 24.0% of SNPs associated with virulence were in LRLD, compared to 37.9% in the whole ST93B SNP population (*P* value < 0.0001) (Table 1). The stronger LD among all SNPs compared to the human infection-related SNPs suggests that the LD is not being driven by virulence-associated forces.

### SNPs in LRLD are highly segregated between ST93A and ST93B

**Fig 3:**
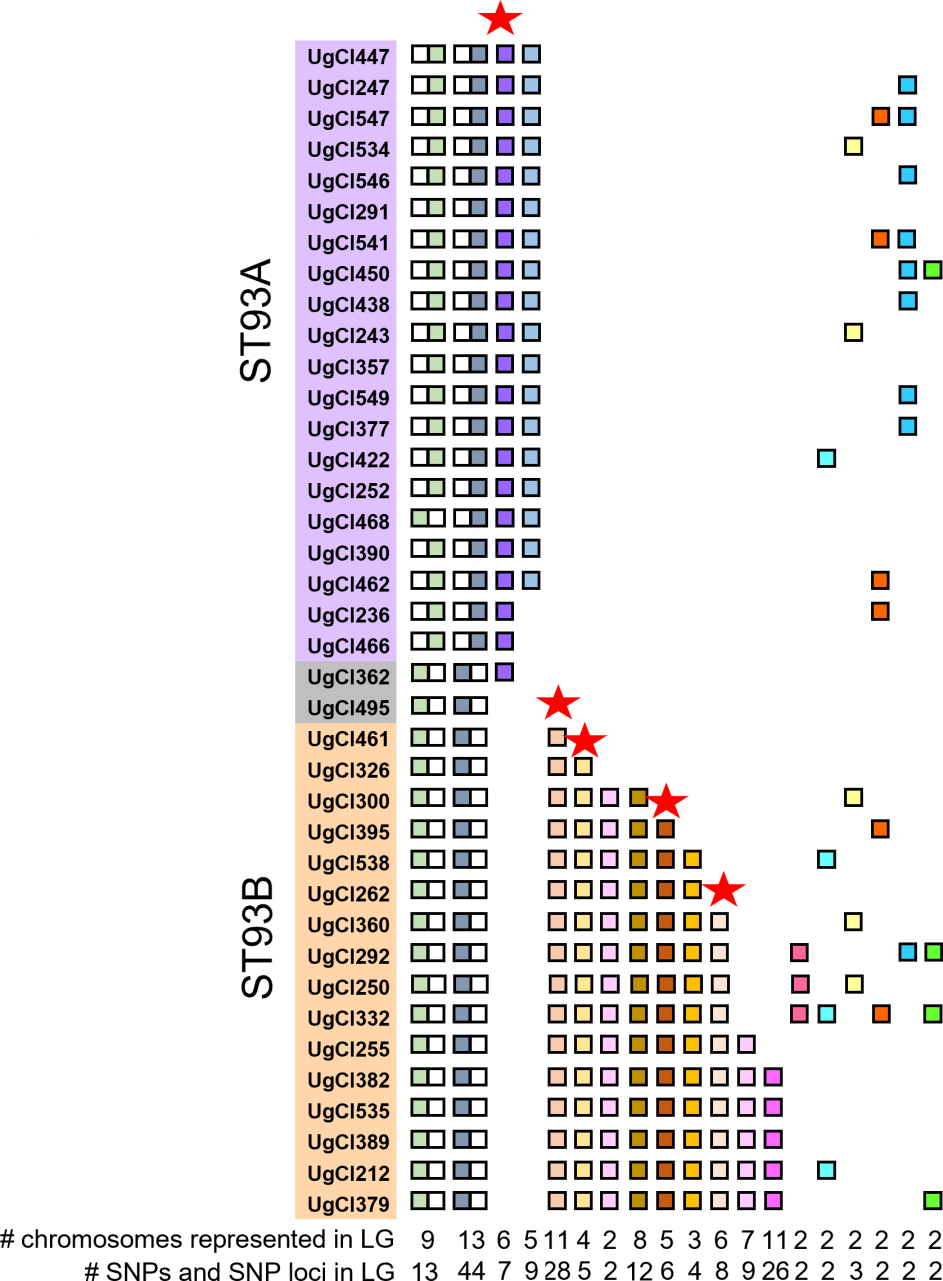
Long-range linkage disequilibrium (LRLD) phylogenetically differentiate ST93A and ST93B. Each linkage group of SNPs in LRLD is represented by a colored box, with location of boxes next to the phylogenetic tree indicating an isolate that contains the linkage group. There was limited overlap between ST93A and ST93B. Red stars indicate linkage groups associated with human disease phenotypes.

To explore the extent to which linkage groups were segregating in one or both subpopulations, we visually examined which LRLD group was found in each isolate (Fig. 3). Some of the larger groups had internal patterns that further segregated ST93A from ST93B and are represented as double boxes; for example, for the first linkage group, 10 SNPs were present in ST93A isolates only (white box on left), but 3 SNPs were present in ST93B isolates only (green box on left), though all were within the same linkage group. We included only linkage groups found in four or more isolates. This analysis revealed a clear pattern of linkage group segregation between ST93A and ST93B (Fig. 3). Of the 19 linkage groups, 14 either differentiated ST93A and ST93B, or were found in only one of the two subpopulations. Most linkage groups specific to a subpopulation were found in ST93B (Fig. 3). Only five linkage groups, all of them rare, did not reflect population substructure; however, those five linkage groups contained only two SNPs, as compared to some of the more phylogenetically driven linkage groups, which had as many as 44 SNPs from up to 13 chromosomes (Fig. 3). Finally, we identified linkage groups associated with human infection phenotypes and marked each of those linkage groups with a red star (Fig. 3). Linkage groups associated with human disease phenotypes included genes involved in stress response pathways, the calcineurin pathway, melanin production, the inositol pathway, mating, general cell functions, and growth in cerebral spinal fluid (CSF) (Table S2). In line with our previous data, we saw that SNPs with human infection phenotypes were more often in LD in ST93B than ST93A.

### Chromosomal structure is similar between ST93A and ST93B

**Fig 4:**
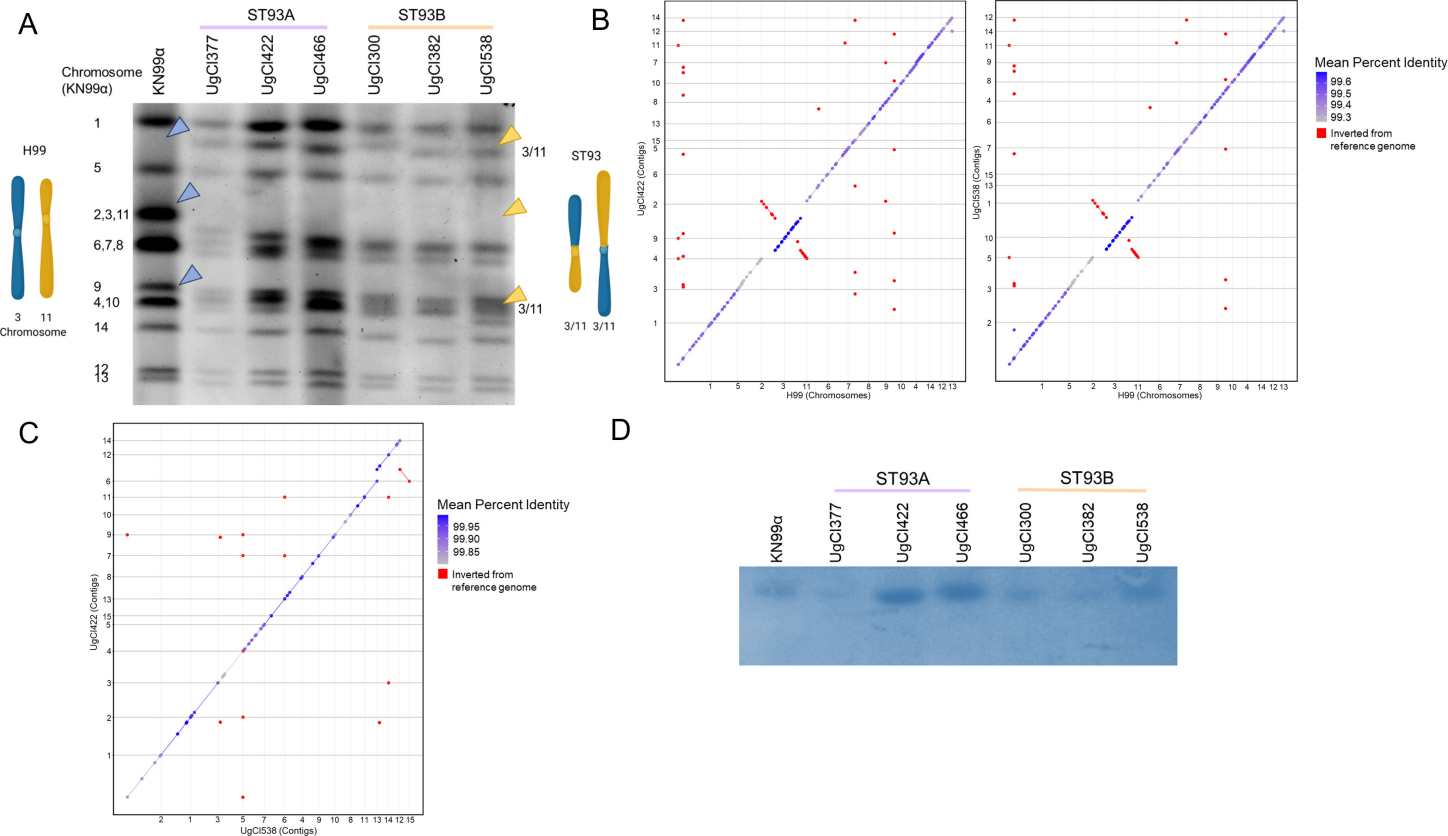
ST93A and ST93B are similar based on chromosome size and structure. (**A**) Contour-clamped homogeneous electric field electrophoresis (CHEF) was used to show the karyotypes of six ST93 isolates compared to KN99α. Chromosomal rearrangements are indicated with arrowheads. Diagrams show chromosomes 3 and 11 from H99 (left) and ST93 isolates (right). (**B**) Long-read sequencing was performed using an Oxford Nanopore MinION sequencer. MUMmer plots were used to compare the H99 genome sequence to an ST93A (UgCl422) and to an ST93B (UgCl538) genome. (**C**) An ST93A genome was additionally compared to an ST93B genome. (**D**) Southern blot from gel shown in (A). Probe encompassed the linkage group on chromosome 2 of H99.

The linkage group segregation between ST93A and ST93B suggested some barrier to recombination, as recombination would have led to more SNP admixture between the subpopulations. Clinical isolates from patients are well known to exhibit structural variations (22, 24–27) and so we hypothesized that ST93A and ST93B may have chromosomal structural variations that hinder recombination. To test this hypothesis, we used contour-clamped homogeneous electric field (CHEF) electrophoresis to investigate the karyotypes of all 38 isolates and 4 non-ST93 isolates (Fig. S1). In general, the ST93 isolates had a different chromosomal structure than the KN99α control. An especially notable difference between the karyotypes of ST93 and KN99α was that, where KN99α had a trimer corresponding to the size of chromosomes 2, 3, and 11, ST93 isolates had a slightly smaller singleton band (Fig. 4A). The ST93 isolates also had two additional chromosomes that were not the same size as any KN99α chromosomes (Fig. 4A). The non-ST93 isolates had an altered karyotype compared to either KN99α or the ST93 isolates. KN99α is very distantly related to the ST93 population (7), so differences in the chromosomal structure were expected. Six ST93 isolates had chromosomal variations from the other ST93 isolates (Table S3). Interestingly, the variations were all unique and not repeated between isolates. Two variations were seen in ST93A isolates and four in ST93B isolates (Table S3).

To further understand chromosomal variation among ST93 isolates, we performed long-read sequencing on the 38 ST93 isolates using an Oxford MinION Nanopore sequencer. The 38 genomes, each of which was sequenced to 24× coverage or above, had genome sizes of around 19 million base pairs (Table S3). The number of contigs ranged from 17 to 28; as the *C. neoformans* H99 reference genome has 14 chromosomes, this was as expected if we assume many contigs did not span the highly repetitive centromere (Table S3). When we compared the ST93 isolates to the H99 control, we observed a chromosome 3, chromosome 11 rearrangement (Fig. 4B), which was identified previously (28–30) and appears to be specific to the H99 lineage. The ST93 isolates had remarkably similar chromosomal structures compared to each other, regardless of subpopulation (Fig. 4C). Across the ST93 population, there were six large chromosomal changes and five smaller chromosomal misalignments (Table S3) Seven of these variations were seen in ST93B isolates and four in ST93A isolates. Importantly, each of these variations was unique to a single isolate and was not repeated in any other isolate, suggesting these variations are unlikely to prevent recombination. Overall, these data suggested that ST93A and ST93B isolates have similar chromosomal structures and so there is not a genomic barrier to recombination that could explain the patterns of linkage disequilibrium.

### Southern blot confirmed the genomic location of linkage groups

The location of SNPs was based on the H99 reference genome. While the karyotyping and long-read sequencing suggested that the physical location of SNPs was in the same location as in the H99 reference genome (except for SNPs on chromosomes 3 and 11), we wanted to confirm that the SNPs in the long-range linkage groups were on the hypothesized chromosomes. To test this, we selected a linkage group found in all ST93A but no ST93B isolates (Table 2). Probes were designed from the H99 genome to bind the region containing SNPs from the linkage group predicted to be on chromosome 2. Southern blotting on six isolates (three from ST93A and three from ST93B) showed that SNPs in the same linkage group were on the same chromosome as in the KN99α reference strain (Fig. 4D). These data suggest the observed LRLD is not due to changes in the genome structure that co-localize SNPs.

**TABLE 2:** Linkage group probed for Southern blot

Chromosome	SNP position	Reference	Alternative
**2^*a*^**	**697983**	**G**	**A**
2	983795	C	G
2	1225303	AG	A
5	276722	G	A
5	1210544	TG	T
11	547884	C	T
14	383626	G	A
14	852745	G	T
10	387430	AGATGACGAGCCGGAT	A

^*a*^ Region captured by the Southern blot probe.

## DISCUSSION

It remains an open question why *Cryptococcus* is a pathogen and how *Cryptococcus* isolates were able to evolve virulence traits. The accidental pathogen hypothesis presents *C. neoformans* as a soil organism that is able to survive in mammalian hosts due to a collection of traits that overlap between survival in the environment and survival in a human host (31). However, *Cryptococcus* sp. can manipulate the host immune response in vertebrates, skewing the immune response toward a type 2 response that prevents fungal clearance and promotes latency/disease (32–34) and environmental isolates tend to be less pathogenic and thermotolerant than clinical isolates (35, 36). These data suggest that there are more complex evolutionary processes occurring that prime an isolate for virulence capability. LD is a valuable tool that can provide insight into population structure and the evolutionary patterns of mutations (37). Using LD, we were able to show that there were SNPs that were extensively linked to phylogeny, as well as SNPs that were recurrent and appeared to have evolved independently. Fascinatingly, the SNPs associated with human disease phenotypes that were identified in a previous GWAS were significantly more likely to be independent SNPs that were unrelated to phylogeny. Previous genomic studies in *C. neoformans* suggested that small genetic variations in key genes appear to be important in altering strain-specific virulence (20–22). The data presented here provide a framework for the evolution of those SNPs: recurrent SNPs that evolve congruently and are selected for during infection, but unrelated to population structure. The presence of recurrent SNPs further highlights the importance of small mutations in specific virulence-related genes.

Our LD analysis provided insight into the ST93 population and subpopulation structure. We had previously observed two subpopulations in ST93: ST93A and ST93B (21). The LRLD linkage group segregation between ST93A and ST93B suggests that ST93A and ST93B are not recombining. Recombination between ST93A and ST93B would lead to admixture of the linkage groups rather than the observed highly segregated SNPs and linkage groups. SNPs in ST93A are not highly structured and most ST93A SNPs and SNP loci are not in LRLD. There are several reasons that the extent of linkage disequilibrium may vary between the two subpopulations; ST93B could have experienced a recent population bottlenecking, there may be cryptic barriers to recombination within ST93B, or selection may have dragged along neutral SNPs associated with a selected SNP that has recently increased in frequency (37).

We examined the karyotypes of isolates from ST93A and ST93B to determine chromosomal differences that might act as a barrier for recombination. Other studies showed that clinical isolates commonly have structural variations and chromosomal changes, but the association between structural variation and virulence remains unclear (22, 26, 28–30, 38). We were unable to find chromosomal differences that might account for a barrier to recombination between the two subpopulations; thus, the LRLD pattern is most likely caused by some other factor. As *C. neoformans* does not mate often in the environment, the subpopulation segregation might be as simple as decreased access to a mating partner (39). Alternatively, the populations may be geographically separated. Unfortunately, there is no data on environmental samples in Uganda, the country where the clinical isolate population was obtained. Even if that data were available, it is difficult to track patient exposure to specific isolates of *C. neoformans* as the initial exposure may have occurred when the patient was very young (1, 2).

The potential virulence implications of the ST93A and ST93B population split are difficult to parse. ST93B has a more structured population and has a non-significant trend toward increased virulence in humans (21) but analysis of a larger population of clinical isolates will be required to determine if ST93B influences patient outcome. Overall, however, this study demonstrates that virulence is more related to independent SNPs rather than population structure. The SNPs associated with human infection phenotypes were not the SNPs that drove the ST93A and ST93B population spit, but rather recurrent SNPs in specific genes. It is possible that SNPs that occur independently in key genes are those that alter the virulence fate of that specific *C. neoformans* isolate. Rather than these mutations important to virulence being collected over long-term evolution, the mutations may instead occur independently during infection. As we identify genes more likely to be mutated under the conditions of infection, these genes will be fundamental for understanding *C. neoformans* virulence.

## MATERIALS AND METHODS

### Linkage analyses

The SNPs used in the linkage analysis were those previously identified as effect SNPs (21). These SNPs were identified by the following criteria: (i) variant was present in the ST93 clade and absent in other ST clades, (ii) variant was present in at least four ST93 strains, (iii) variant was non-synonymous and located outside extreme telomeric or centromeric regions. Linkage disequilibrium was then calculated as *r*^2^, the square of the correlation between alleles at two polymorphic loci, for each pair of SNPs for which the minor allele was present at a frequency >0.1 (MAF >0.1). *r*^2^ = *D*^2^ / [*pA*(1 *pA*)*pB(1*−pB*)], where *D* = *pAB* pApB, *pAB* is the frequency of *AB* genotypes, *pA* is the frequency of the *A* allele at one locus, and *pB* is the frequency of the *B* allele at a second locus (40). Values of *r*^2^ = 0 indicate that alleles at the two loci are inherited independently, whereas |*r*^2^| = 1 indicates the alleles at the two loci are inherited together (i.e., chromosomes with *A* will also have *B*). SNPs were then grouped into LD groups, in which the SNPs in a group had *r*^2^ > 0.95. Because the samples came from two distinct populations (21), much of the LD found in the entire data set may reflect population structure, something that was confirmed in preliminary analyses. Data were separated into ST93A and ST93B and LD was calculated separately. LD groups were formed within each population using the same criterion as above, that is, *r*^2^ > 0.95.

### CHEF electrophoresis and Southern blot

To make agar plugs for the CHEF electrophoresis, isolates were grown overnight in yeast nitrogen base (YNB) supplemented with 1 M NaCl, pelleted by centrifugation, then washed in 0.5 M NaCl with 50 mM EDTA. Cells were embedded into agar and simultaneously lysed with Zymolyase 20T (MP Biomedicals, Irvine, CA). Plugs were solidified and washed overnight in 500 mM EDTA and 10 mM Tris. A solution containing 5 mg/mL proteinase K and 5% sarcosyl was added, the plugs were incubated at 50°C for 5 h, and then washed overnight in 20 mM Tris and 50 mM EDTA. Plugs were washed once in 20 mM Tris and 50 mM EDTA with phenylmethylsulfonyl fluoride, then two times with 2 mM Tris and 1 mM EDTA, and finally stored at 4°C. Agar plugs were analyzed on a CHEF apparatus (Bio-Rad, Hercules, CA), using the following program: initial A time 75 s and final A time 150 s for 30 h; initial B time 200 s and final B time 400 s for 60 h. Southern blotting was performed as described previously (41). In short, an imaged CHEF gel was nicked using a Stratalinker (Statagene, La Jolla, CA) and denatured. DNA was transferred overnight to a nitrocellulose membrane. The DNA was crosslinked to the membrane using a Stratalinker and the blot was hybridized with a DIG-labeled probe (Roche, Basel, Switzerland) that bound to a region on chromosome 2 and imaged.

### Long-read sequencing

DNA was extracted using cetyltrimethylammonium bromide (CTAB) as described previously (42), with the following changes. Each isolate was grown overnight in replicate 50 mL yeast extract peptone dextrose (YPD) broth cultures supplemented with 0.001% chloramphenicol. The cultures were centrifuged and then lyophilized overnight in a 50-mL Falcon tube. Glass beads were added to each tube; lyophilized pellets were dispersed by vortexing for 20 min on a multitube vortex (Benchmark Scientific Inc, Sayreville, NJ). CTAB solution was added to each tube and any remaining pellet was dispersed with a sterile pipet tip. Subsequent vortex steps were performed by hand on a single-tube vortex. Each Falcon tube was incubated at 65°C, chloroform added, and centrifugation performed. The supernatant was transferred to a new tube, isopropanol added, and tubes were mixed by gentle rotation by hand until DNA was visible. DNA was removed from the tube using a glass rod, added to 1 mL ethanol, and centrifuged for 5 min. Ethanol was removed after centrifugation and pellets were dried by heating at 37°C. Sterile water and RNase A were added and the tube was incubated at 37°C until the DNA was fully dissolved. DNA was quantified using a Nanodrop (ThermoFisher Scientific, Waltham, MA), then immediately prepared for long-read sequencing.

Libraries were prepared using an Oxford Nanopore Rapid Barcoding Kit (Oxford Nanopore, Oxford Science Park, UK). Each library contained six isolates. About 2.5 µL of each freshly isolated DNA sample was added and each isolate was assigned two barcodes. The library was sequenced using an Oxford Nanopore flow cell (R9) and MinION sequencer (Mk1b). Each sequencing run was 72 h. After 18 h, the sequencing output was analyzed and the flowcell was washed using a Flow Cell Wash Kit (EXP-WHS003 Oxford Nanopore) and a fresh library was loaded, as needed.

### Sequence assembly

Raw long-read sequences from the Minion sequencer were basecalled and demultiplexed using Guppy 4.0.5. Quality assessment of the fastq files was performed using NanoPlot 1.40.0. The raw long reads were assembled using the *de novo* assembler Canu 2.2 (43), which is designed to handle high-noise and high-error long-read data and incorporates read trimming, error correction, and consensus calling. The parameters used: genomeSize = 19 mb (based on the size of the reference genome, H99), correctedErrorRate = 0.075, minReadLength = 700, minOverlapLength = 500 minInputCoverage = 5, and correctedErrorRate = 0.144. The assembled genomes were polished with Illumina paired end sequences using Nextpolish 1.4.0 with the following parameters: sgs_options = -max_depth 100, lgs_options=-min_read_len 2 k, max_depth 80, and lgs_minimap2_options = -x map-ont (44). The quality of the assembled genome was assessed using the depth of coverage, number of contigs, and genome completeness. Genome assemblies were kept if the depth of coverage was *X*≥24 and the number of contigs ≤30. The completeness of the assemblies was evaluated using BUSCO 2.0 (Benchmarking Universal Single-Copy Orthologs) (45). All assemblies had BUSCO scores of ≥97%.

### Whole-genome alignment

The MUMmer 4.0.0beta2 pipeline, NUCmer was used to align the *de novo* assembled whole genomes against the H99 reference genome (GCA_000149245.3) (46). The delta file from NUCmer was filtered with the following parameters: -i 98 and -l 5,000 bp to keep alignments with 98% similarity per 5,000 bp. The filtered delta file was then used as input for the mummerplot utility with the -l, --fat and --png options.

## Data Availability

All data and scripts are available at GitHub at https://github.com/dkisakye/cryptococcus-neoformans-long-read-denovo-assembly.git. Sequence data are available at NCBI under BioProject ID PRJNA1141514.

## SUPPLEMENTAL MATERIAL

Fig. S1 spectrum.01709-24-s0001.pdf CHEF karyotypes for all 38 ST93 isolates and five non-ST93 isolates.

Table S1 spectrum.01709-24-s0002.xlsx Genes in linkage groups associated with human infection phenotypes.

Table S2 spectrum.01709-24-s0003.xlsx Genes in linkage groups associated with human infection phenotypes and putative role in virulence.

Table S3 spectrum.01709-24-s0004.xlsx Long-read sequencing statistics and structural variation.

ASM does not own the copyrights to Supplemental Material that may be linked to, or accessed through, an article. The authors have granted ASM a non-exclusive, world-wide license to publish the Supplemental Material files. Please contact the corresponding author directly for reuse.
