# Erratum for Jackson et al., “Virulence-associated variants in *Cryptococcus neformans* sequence type 93 are less likely to be associated with population structure compared to independent rare mutations”

**DOI:** 10.1128/spectrum.00525-25

**Published:** 2025-03-17

**Authors:** Katrina M. Jackson, Kisakye Diana Kabbale, Marissa Macchietto, David Meya, Peter Tiffin, Kirsten Nielsen

## ERRATUM

Volume 13, no. 1, e01709-24, 2025, https://doi.org/10.1128/spectrum.01709-24. Page 11, Acknowledgments: “R01AI34636” should read “R01AI176922.”

