# Supplementary material for: Virulence-associated variants in Cryptococcus neoformans sequence type 93 are less likely to be associated with population structure compared to independent rare mutations
Source: Microbiol Spectr. 2024 Nov 27;13(1):e01709-24. doi: 10.1128/spectrum.01709-24 (PMC11705857; doi:10.1128/spectrum.01709-24)
Supplement: Fig. S1 — CHEF karyotypes for all 38 ST93 isolates and five non-ST93 isolates. [file spectrum.01709-24-s0001.pdf]

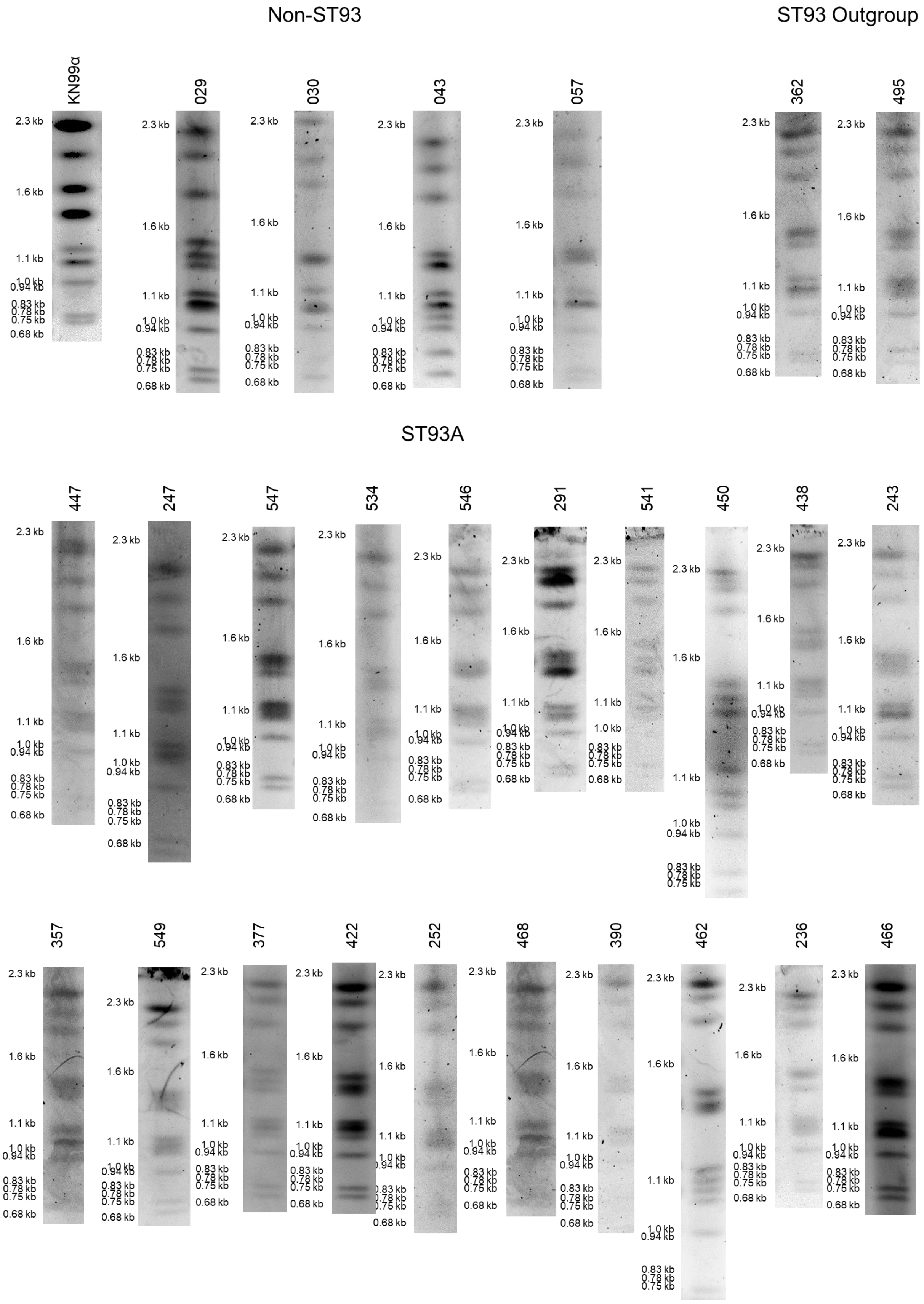
Non-ST93
ST93 Outgroup
KN99α
029
030
043
057
362
495
ST93A
447
247
547
534
546
291
541
450
438
243
357
549
377
422
252
468
390
462
236
466
2.3 kb
1.6 kb
1.1 kb
1.0 kb
0.94 kb
0.83 kb
0.78 kb
0.75 kb
0.68 kb

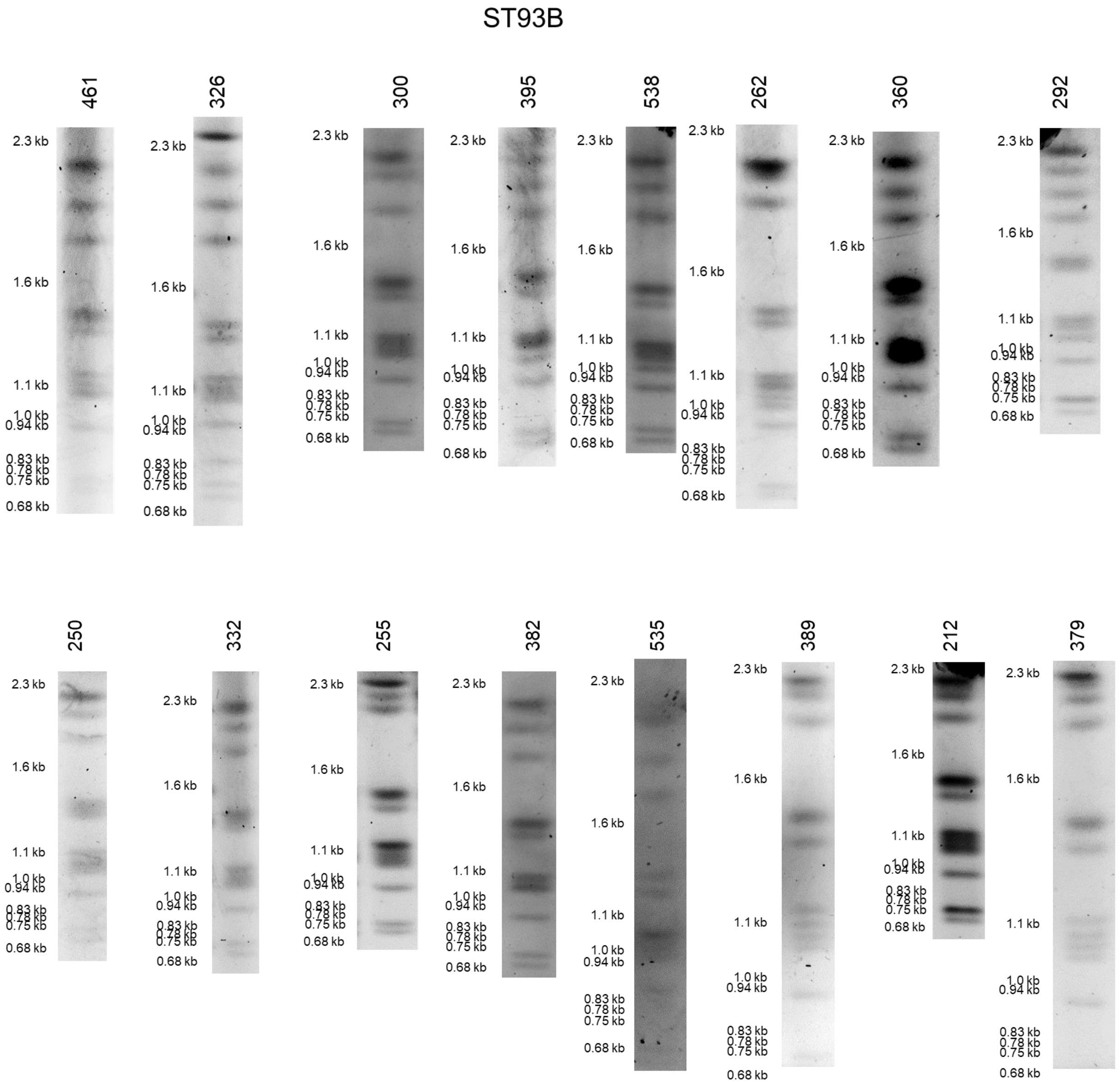

Supplemental figure 1: CHEF karyotypes for all 38 ST93 isolates and five non-ST93 isolates. Contour-clamped homogeneous electric field electrophoresis was used to show the karyotypes of all isolates the ST93 population and five non-ST93 isolates, including KN99α.
